# Minimally invasive system to reliably characterize ventricular electrophysiology from living donors

**DOI:** 10.1038/s41598-020-77076-0

**Published:** 2020-11-17

**Authors:** Aida Oliván-Viguera, María Pérez-Zabalza, Laura García-Mendívil, Konstantinos A. Mountris, Sofía Orós-Rodrigo, Estel Ramos-Marquès, José María Vallejo-Gil, Pedro Carlos Fresneda-Roldán, Javier Fañanás-Mastral, Manuel Vázquez-Sancho, Marta Matamala-Adell, Fernando Sorribas-Berjón, Javier André Bellido-Morales, Francisco Javier Mancebón-Sierra, Alexánder Sebastián Vaca-Núñez, Carlos Ballester-Cuenca, Miguel Ángel Marigil, Cristina Pastor, Laura Ordovás, Ralf Köhler, Emiliano Diez, Esther Pueyo

**Affiliations:** 1 Biomedical Signal Interpretation and Computational Simulation (BSICoS) Group, Aragón, Institute of Engineering Research (I3A) and Instituto de Investigación Sanitaria (IIS) Aragón, University of Zaragoza, Edificio I+D+i, C/Mariano Esquillor s/n, 50018 Zaragoza, Spain; 2 Department of Cardiovascular Surgery, University Hospital Miguel Servet, Zaragoza, Spain; 3 Department of Pathology, San Jorge Hospital, Huesca, Spain; 4 Aragón Institute of Health Sciences (IACS), Zaragoza, Spain; 5 Aragón Agency for Research and Development (ARAID), Zaragoza, Spain; 6 Institute of Experimental Medicine and Biology of Cuyo (IMBECU), CONICET, Mendoza, Argentina; 7 Biomedical Research Networking Center in Bioengineering, Biomaterials and Nanomedicine (CIBER-BBN), Zaragoza, Spain

**Keywords:** Cell biology, Molecular biology, Cardiology

## Abstract

Cardiac tissue slices preserve the heterogeneous structure and multicellularity of the myocardium and allow its functional characterization. However, access to human ventricular samples is scarce. We aim to demonstrate that slices from small transmural core biopsies collected from living donors during routine cardiac surgery preserve structural and functional properties of larger myocardial specimens, allowing accurate electrophysiological characterization. In pigs, we compared left ventricular transmural core biopsies with transmural tissue blocks from the same ventricular region. In humans, we analyzed transmural biopsies and papillary muscles from living donors. All tissues were vibratome-sliced. By histological analysis of the transmural biopsies, we showed that tissue architecture and cellular organization were preserved. Enzymatic and vital staining methods verified viability. Optically mapped transmembrane potentials confirmed that action potential duration and morphology were similar in pig biopsies and tissue blocks. Action potential morphology and duration in human biopsies and papillary muscles agreed with published ranges. In both pigs and humans, responses to increasing pacing frequencies and β-adrenergic stimulation were similar in transmural biopsies and larger tissues. We show that it is possible to successfully collect and characterize tissue slices from human myocardial biopsies routinely extracted from living donors, whose behavior mimics that of larger myocardial preparations both structurally and electrophysiologically.

## Introduction

Management of cardiovascular diseases poses a great challenge for healthcare systems. Improved understanding of the physiology and pathophysiology of the complex and highly heterogeneous human heart requires integrated multi-level analysis able to account for spatial and temporal variability in cardiac behavior^1–4^. Although extensive research has been conducted in animals, the relevance to humans remains largely unknown. This can be, to a large extent, attributed to the scarce access to human cardiac tissue samples and the low-throughput research on whole organs.

Cardiac tissue slices preserve the complex three-dimensional structure, multicellularity and interactions between cell types in the heart^5–9^. Thin tissue slices can benefit from oxygen and metabolic substrate diffusion, allowing its function to be maintained without the need for coronary perfusion^10,11^. Organotypic slices have been shown to constitute an affordable model to investigate structure^5^, metabolism^11^ and function^12–14^ of cardiac tissue and its response to pharmacological and toxicological interventions^6,7,15,16^. New systems and protocols have been developed that allow biomimetic culture of cardiac tissue slices for several days^17–20^.

In humans, however, the availability of cardiac ventricular samples is mostly limited to trabeculae or papillary muscles from intracardiac ablation procedures^7,15,21,22^ and to a reduced number of explanted failing hearts or hearts from organ donors not transplanted for technical reasons^6,11,15,16,19,20,23,24^. There is a need for a higher throughput system that allows the characterization of ventricular tissue electrophysiology in health and disease, with a representation of inter- and intra-individual variability in the spatial and temporal domains^23,25–28^.

We propose full-thickness transmural core biopsies to obtain small human ventricular samples from living donors, compatible with routine cardiac surgical procedures. A 14 gauge (G) core biopsy needle allows surgeons to perform a biopsy in an easy and safe way, on minimal time, with limited bleeding and without organ damage^29–32^. In addition, the collection with this type of biopsy needle is highly reproducible, regarding sample size and morphology, and allows the study of different layers of the ventricular wall.

Here, we evidence that transmural core biopsies are suitable for robust and accurate electrophysiological characterization of the ventricular myocardium. In pigs, we compare the electrophysiology of myocardial slices from transmural core biopsies and transmural ventricular blocks^6,7,12,16,33^ obtained from the same ventricular region. In humans, we characterize electrophysiological signals recorded from slices of transmural core biopsies and papillary muscles of living donors. In all cases, we assess the response to an increase in the pacing frequency and to the administration of the β-adrenergic agonist isoproterenol.

## Methods

Detailed methods are available in the Supplementary Material.

### Myocardial tissue collection

**Figure 1:**
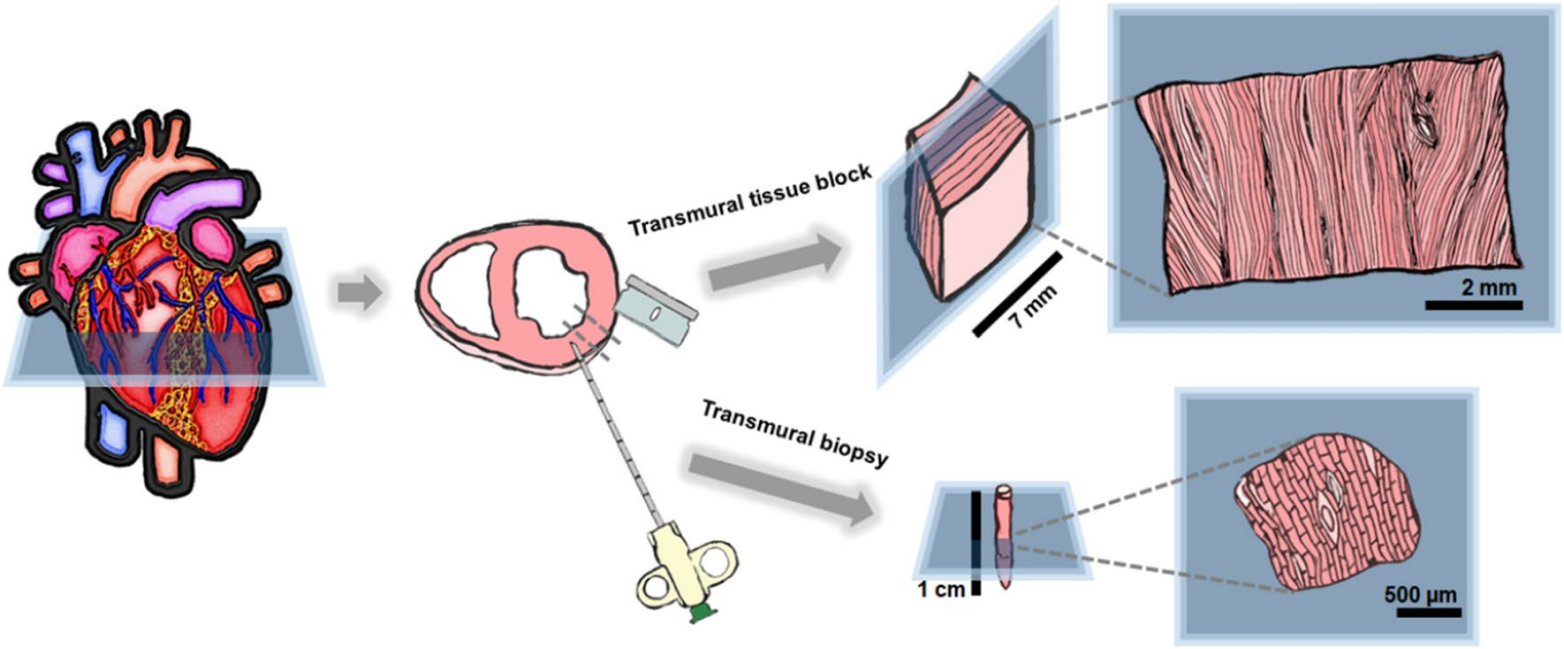
Scheme of tissue collection and preparation. Transmural tissue blocks and transmural core biopsies from a neighboring left ventricular region obtained from pig hearts by using a razor blade and a transmural core biopsy needle, respectively. Slice sectioning in planes parallel to the epicardium is essential to maximize the amount of longitudinal myocardial fibers.

Porcine left ventricular transmural core biopsies were obtained from 5 pigs after sacrifice by intravenous administration of KCl solution (1 mEq/kg) performed under deep anesthesia with propofol (intravenous administration, up to 6 mg/kg) and inhaled sevoflurane (1.9%). A disposable 14 G tru-cut biopsy needle (Bard Mission 1410MS, Bard) was used to extract transmural core biopsies of approximately 1.2 mm diameter and up to 10 mm long (Fig. 1). Transmural tissue blocks (surface area ≈ 5 × 7 mm) were cut with a single edge razor blade from a neighboring zone (Fig. 1). All animal experiments complied with the regulations of the local animal welfare committee for the care and use of experimental animals and were approved by local authorities (Ethics Committee on Animal Experimentation, CEAEA, of the University of Zaragoza). All animal procedures conformed to the guidelines from Directive 2010/63/EU of the European Parliament on the protection of animals used for scientific purposes.

Human left ventricular transmural core biopsies were collected by experienced cardiothoracic surgeons at Miguel Servet University Hospital. Specimens were obtained from 21 patients undergoing valve replacement surgery or coronary artery bypass grafting. A disposable 14 G tru-cut biopsy needle (Bard Mission 1410MS, Bard) was used to extract one biopsy from every patient during cardiac arrest soon after the patient was placed on cardiopulmonary bypass. Papillary muscles resected during valve replacement from 8 different patients were included for comparison. The clinical characteristics of donors are summarized in Supplementary Table 1. All patients gave written informed consent before surgery and prior to their inclusion in the study. The study conforms to the principles outlined in the Declaration of Helsinki and was approved by the local Ethics Committee (CEICA, reference number PI17/0023).

### Tissue slice preparation

Upon collection, porcine and human tissues were immediately submerged in ice-cold pre-oxygenated Tyrode’s solution. Transport time to the laboratory was less than 10 min for human tissues and less than 1 h for porcine tissues.

Tissue blocks were directly glued onto the vibratome cutting stage, mounting them epicardium-side down to ensure maximum longitudinal alignment of muscle fibers with the slicing plane (Fig. 1). Transmural core biopsies were embedded in 4% low-melting agarose (Roth, Karlsruhe, Germany) and glued onto the vibratome cutting stage with the biopsy in upright position (Fig. 1) to be sliced parallel to the epicardial plane. Papillary muscles were sliced with the chordae tendinae aligned with the slicing plane.

Tissue blocks, transmural core biopsies and papillary muscles were cut in ice-cold pre-oxygenated Tyrode’s solution at a thickness of 350 µm, employing a high precision vibratome (Leica VT1200S, Leica Microsystems, Germany).

After slicing, sections were paraformaldehyde-fixed for histological evaluation or kept at room temperature in pre-oxygenated BDM-Tyrode’s solution for electrophysiological analysis by optical mapping within the following 8 h and staining for viability assessment.

See Supplementary Material for a detailed description of the procedures.

### Optical mapping of transmembrane potential

Myocardial tissue slices were optically mapped with a MiCAM O5-Ultima CMOS camera (SciMedia, Costa Mesa, CA). Further details are available at Supplementary Material.

For transmembrane potential measurements, slices were incubated at room temperature with the excitation–contraction uncoupler blebbistatin (10 µM, Tocris Bioscience, St. Louis, MO) for 30 min and with the voltage-sensitive dye RH237 (Invitrogen, Carlsbad, CA) for 15 min at a concentration of 7.5 µM in pre-oxygenated Tyrode’s solution. After staining, tissue slices were washed in pre-oxygenated Tyrode’s solution for 30 min to 1 h. Optical measurements were conducted in pre-oxygenated Tyrode’s solution while placed in a heated chamber at 35 °C, equipped with two platinum field-stimulation electrodes. 20-s recordings were acquired at pacing frequencies of 1 and 2 Hz after a short period of stimulation to allow slices to adjust to the pacing rate. β-adrenergic stimulation responsiveness was evaluated by application of isoproterenol (100 nM, Sigma Aldrich).

### Optical mapping data analysis

Custom-written software was developed for optical mapping data analysis (MATLAB R2017a, The MathWorks Inc., Natick, MA). See Supplementary Material for a detailed description of the analyzed samples.

Optical action potential (AP) signals were high-pass filtered (0.4 Hz cut-off frequency) to remove baseline drift corresponding to frequencies smaller than 0.4 Hz and subsequently filtered by an adaptive spatio-temporal Gaussian filter^34^. AP duration (APD) was calculated by measuring the elapsed time between the activation, defined as the time occurrence of the maximum AP upslope, and the time for 80% repolarization. APD and activation time maps of the myocardial slices were generated for the whole set of pixels^35^. A signal-to-noise ratio (SNR) value was calculated for each of them as the AP amplitude divided by the root mean-square of voltage during the diastolic interval^36^. A threshold on SNR was set and APD and activation maps were presented only for pixels with SNR above it.

Relative APD values measured after β-adrenergic stimulation or after increasing the stimulation frequency to 2 Hz were calculated as normalized with respect to those measured at baseline while pacing at 1 Hz.

### Statistical analysis

Quantitative data are presented as median [first quartile (Q1)-third quartile (Q3)], or as averaged values for percentages of cases. The notation n/N is used to denote n slices from N tissues (either tissue blocks, papillary muscles, or transmural core biopsies). In the viability evaluation by confocal microscopy imaging, the notation i/n/N denotes i images of different areas from n slices from N tissues. When optical mapping measurements are presented from different pixels across each slice, the notation p/n/N is used to denote p pixels from n slices from N tissues.

The effects of β-adrenergic stimulation and of increased pacing frequency on APD were assessed by using the non-parametric Wilcoxon signed rank test for paired samples, as the data were not normally distributed according to Shapiro–Wilk test. To compare normalized APD values between the groups of measurements from tissue blocks/papillary muscles and the group of measurements from transmural core biopsies, the non-parametric Mann–Whitney U-test for unpaired samples was used.

A *p* value < 0.05 was considered as statistically significant.

## Results

### Integrity of tissue slices

**Figure 2:**
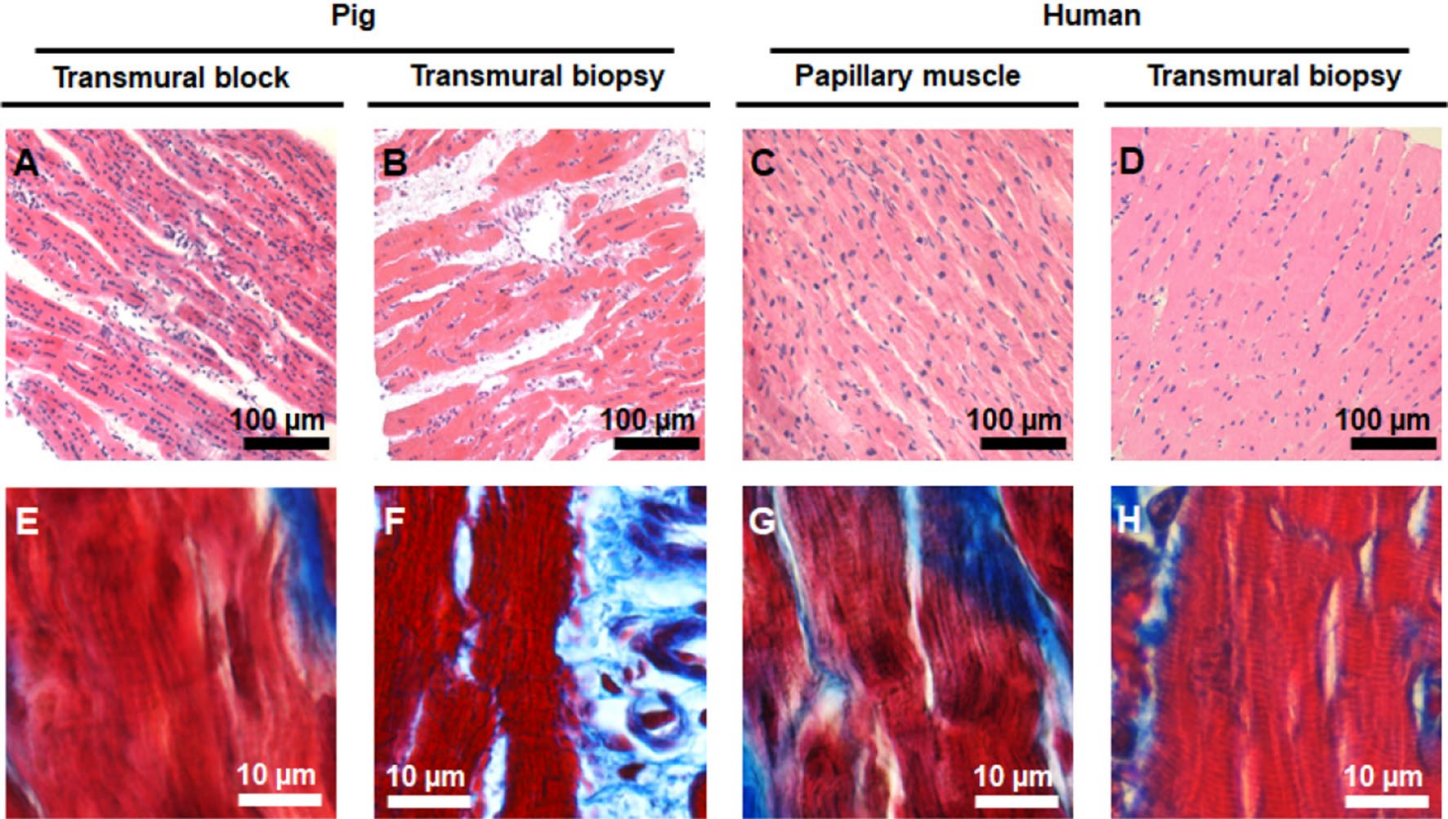
Histological evaluation of pig and human ventricular tissue specimens showing preserved physiological morphology and structure. Representative photomicrographs of pig tissue block slices (**A**,**E**), pig transmural core biopsy slices (**B**,**F**), human papillary muscle slices (**C**,**G**) and human transmural core biopsy slices (**D**,**H**), stained with hematoxylin/eosin and imaged at 20 × magnification (upper panels) or stained with Masson’s trichrome and imaged at 63 × magnification (lower panels). The majority of fibers are longitudinally aligned, with elongated fibroblasts running among them. Note the presence of regular sarcoplasmic cross striations and of blood vessels surrounded by collagenous connective tissue (stained blue in the lower panels) as well the absence of signals of tissue damage like contraction bands or wavy fibers.

We first assessed integrity of vibratome slices by histological analysis. Results are presented in Fig. 2 for porcine transmural tissue blocks (Fig. 2A,E) and transmural core biopsies (Fig. 2B,F) as well as for human papillary muscles (Fig. 2C,G) and transmural core biopsies (Fig. 2D,H). Preserved myocardial structural integrity was demonstrated in all pig and human cases, with the majority of myocardial fibers being longitudinally aligned, as revealed by hematoxylin/eosin staining (Fig. 2A–D), and with regular sarcoplasmic cross striations, evidenced at high magnification by Masson’s Trichrome staining (Fig. 2E–H). Contraction bands or wavy fibers, indicative of tissue damage, were not observed. Elongated fibroblasts in close contact with small capillaries and myocytes could be distinguished together with the presence of blood vessels surrounded by collagenous connective tissue (Fig. 2B,F). Intact cellular organization and retained tissue architecture were equally observed in slices obtained from transmural core biopsies (Fig. 2B,D,F,H) or from larger pieces of cardiac tissue (Fig. 2A,C,E,G) for both pigs and humans. As a side note, an increment in cell size and nuclear size as a typical indicator of hypertrophied myocardium could be observed in the cardiomyocytes of some specimens, like the one shown in panel 2C coming from a patient undergoing mitral valve repair.

In some slices of tissue specimens, we observed areas with both longitudinal and transverse fibers (Supplementary Figure 1A). A few slices showed small areas lacking myocardial fibers. Human papillary muscles showed varying degrees of intercalated connective tissue of the chordae tendinae (Supplementary Figure 1B). Transmural core biopsies of elderly patients or patients with myocardial hypertrophy frequently presented higher levels of interstitial fibrosis. Moreover, in some but not all slices from specific tissue specimens, a region was found to be occupied by part of a coronary artery or a vein and its surrounding connective tissue (Supplementary Figure 1C).

### Viability of tissue slices

**Figure 3:**
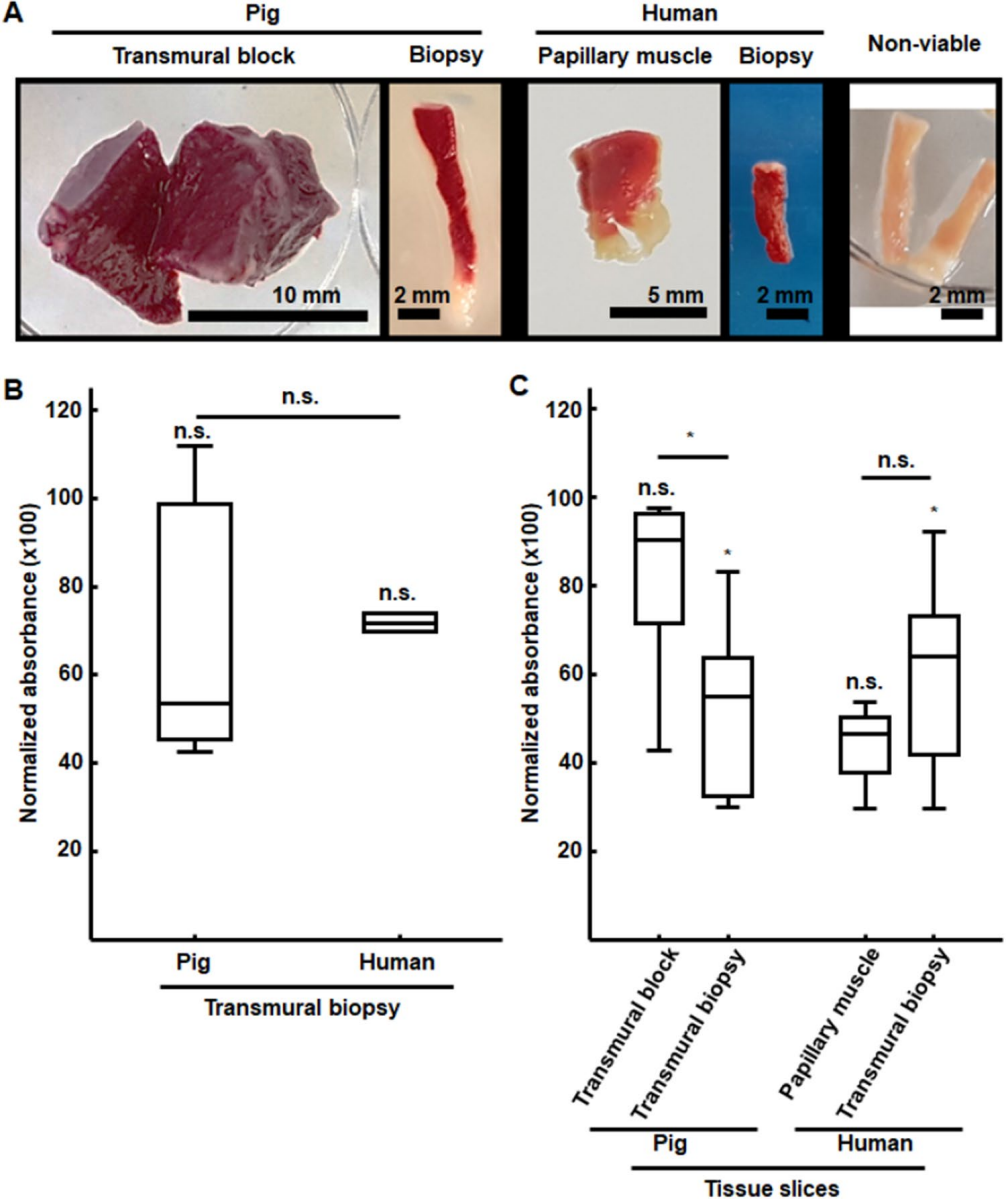
Viability evaluation by colorimetric measurement of enzyme activity. (**A**) Representative images of TTC staining of pig transmural tissue blocks and transmural core biopsies as well as human papillary muscles and transmural core biopsies. Non-viable transmural biopsies (right image) are shown for comparison. All tissues showed widely extended homogeneous deep red staining correlating with viable tissue. (**B**) Quantitative evaluation of viability for transmural core biopsies as relative to tissue blocks in pigs and relative to papillary muscles in humans, with no statistically significant differences in the absolute values in any of the two cases. Differences between relative viabilities in pig and human biopsies were not statistically significant. *n.s.* non-statistically significant differences according to Mann–Whitney U-test. (**C**) Effects of vibratome slicing on tissue viability by quantification of relative viability of the slices with respect to each corresponding tissue. Slicing led to significant differences in absolute absorbance values only for transmural core biopsies. Relative variabilities were statistically significantly different between transmural biopsy slices versus larger tissues for pigs but not for humans. *p < 0.05 and *n.s.* non-statistically significant differences according to Mann–Whitney U-test. n/N = 8/2 for pig tissue blocks, n/N = 104/6 for pig transmural biopsies, n/N = 4/3 for human papillary muscles and n/N = 77/7 for human transmural biopsies.

We next performed TTC staining analysis to assess the optimal preservation of the ventricular tissue specimens after extraction from the heart and subsequent transportation to the laboratory (Fig. 3A). In all cases, we observed an extended homogeneous deep red staining, correlating with viable tissue. Only minor areas of dead tissue in some edges were noted, as in the lower end of the transmural core biopsy and the papillary muscle of the second and third panels in Fig. 3A, likely tore during the extraction from the heart.

A quantitative outcome of the viability of transmural core biopsies with respect to tissue blocks in pigs as well as of transmural core biopsies with respect to papillary muscles in humans is presented in Fig. 3B. The median relative viability of transmural core biopsies was above 50% of that in porcine tissue blocks and above 70% of that in human papillary muscles. Absolute absorbance values of transmural core biopsies were not statistically significantly different between biopsies and larger pieces of tissue or between porcine and human biopsies.

Additionally, we assessed the effects of vibratome slicing and electrophysiological assessment on tissue viability after 4–8 h of optical mapping recordings. The results from the TTC staining analysis of the tissue slices is presented in Fig. 3C. In the porcine transmural tissue blocks, slicing and electrophysiological procedures led to minimal damage, with median relative viability of the slices in relation to the intact tissue block being 90% and no statistically significant differences in absolute absorbance values between the slices and the whole tissues. For human papillary muscles, the median relative viability of the slices was reduced to 47%, probably due to their higher inhomogeneity in the distribution and alignment of myocardial tissue fibers and, again, no slicing-induced significant differences in absolute absorbance values. For porcine and human transmural core biopsies, the median relative viability of the slices was 55% and 64% of that for whole transmural core biopsies in pigs and humans, respectively. In both species, the absolute viability of transmural core biopsies slices was significantly different from that of the intact biopsies, indicating some damage caused by vibratome slicing. When comparing the slicing-induced damage in transmural core biopsies versus the slicing-induced damage in larger pieces of tissue, this was found to be significantly larger in pigs but not in humans.

**Figure 4:**
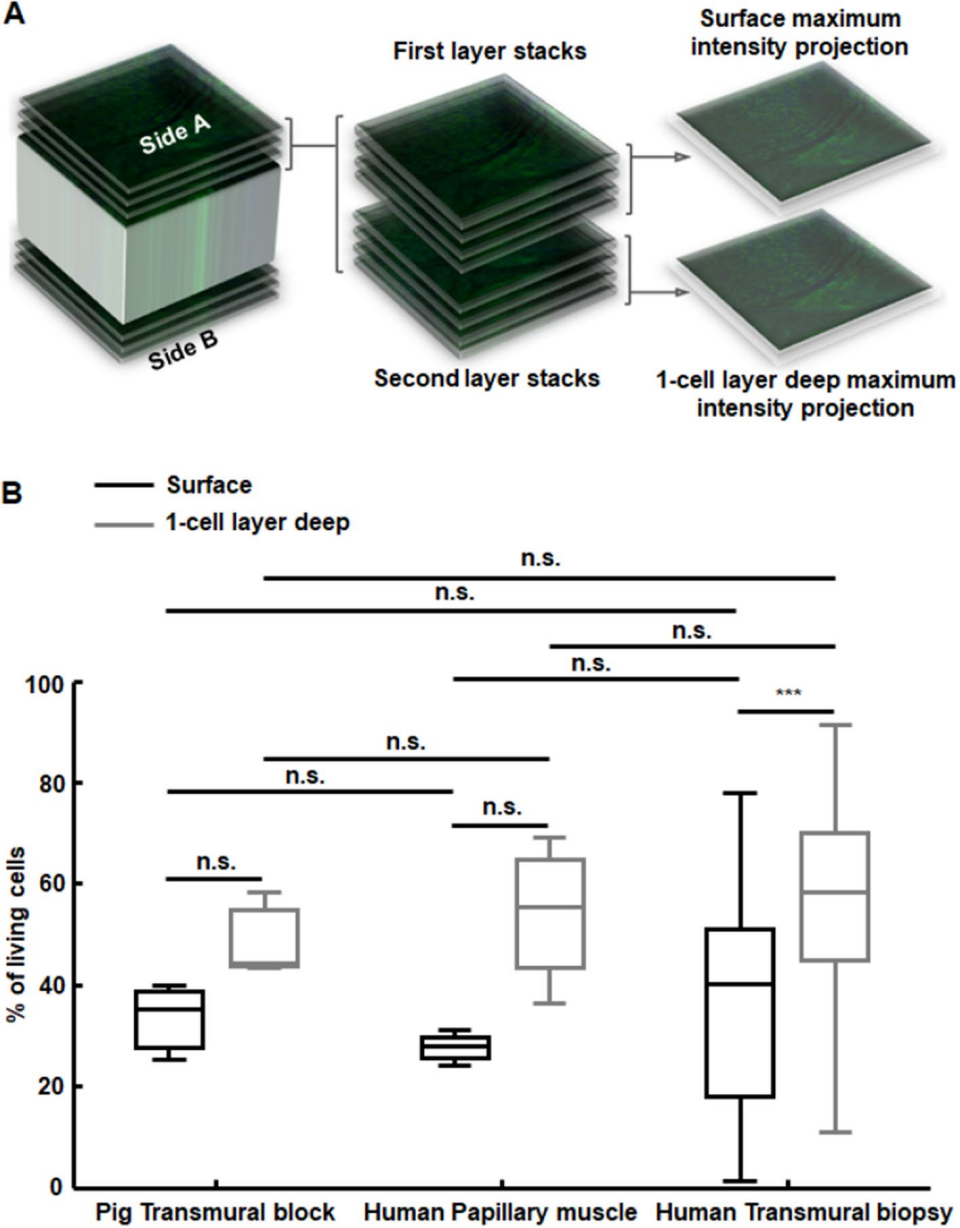
Viability evaluation by vital staining with Dapi/Syto9 and confocal microscopy imaging. (**A**) Processing scheme. Z-series of images were collected at 50–150 longitudinal focal planes at the central core of both sides of the slices. Z-stacks from each side were divided into an external and an internal subset and each one compressed into a single image to evaluate the percentage of damaged cells. (**B**) Boxplots showing percentages of viable cells in the surface (black boxes) and in the first cell layer directly below the surface (grey boxes). i/n/N = 3/1/1 for pig tissue block, i/n/N = 4/2/2 for human papillary muscles and i/n/N = 24/12/12 for human transmural biopsies. ***p < 0.001 and *n.s.* non-statistically significant differences either in the comparison between external and inner layers (Wilcoxon signed rank test for paired samples) or between different tissues types (Mann–Whitney U-test for unpaired samples).

Further assessment of vibratome slices’ viability by Dapi/Syto9 staining and confocal microscopy analysis is presented in Fig. 4. Due to laser penetration limitations, viability results are compared for the first half and second half portions of the outer layers of cardiomyocytes in the tissue slices. In the first most external portion of the slices from pig tissue blocks, human papillary muscles and human transmural core biopsies, the median percentages of viable cells were 35%, 28% and 40%, which increased to 44%, 56% and 58% respectively for the second half portion. This increment was statistically significant for human transmural slices. Provided the observed increase in viability when going deep into the tissue slice, viability percentages up to 100% can be expected for inner layers in the central zone of the slices (in accordance to viability assessment in the whole slices with the TTC enzymatic method described in Fig. 3C). It should be noted that median percentages of viable cells were similar for the slices of all analyzed types of tissues, and even slightly larger for transmural core biopsies, thus indicating feasibility of biopsy slices for electrophysiological analysis.

### Optical mapping of pig tissue slices

Viable pig slices for electrophysiological analysis were obtained from all tissue blocks and all transmural core biopsies. For each block or biopsy, more than 15 slices were generally obtained. In the case of the tissue blocks, 100% of the vibratome slices rendered electrophysiological signals, with the corresponding percentage in the transmural core biopsies being 70%.

**Figure 5:**
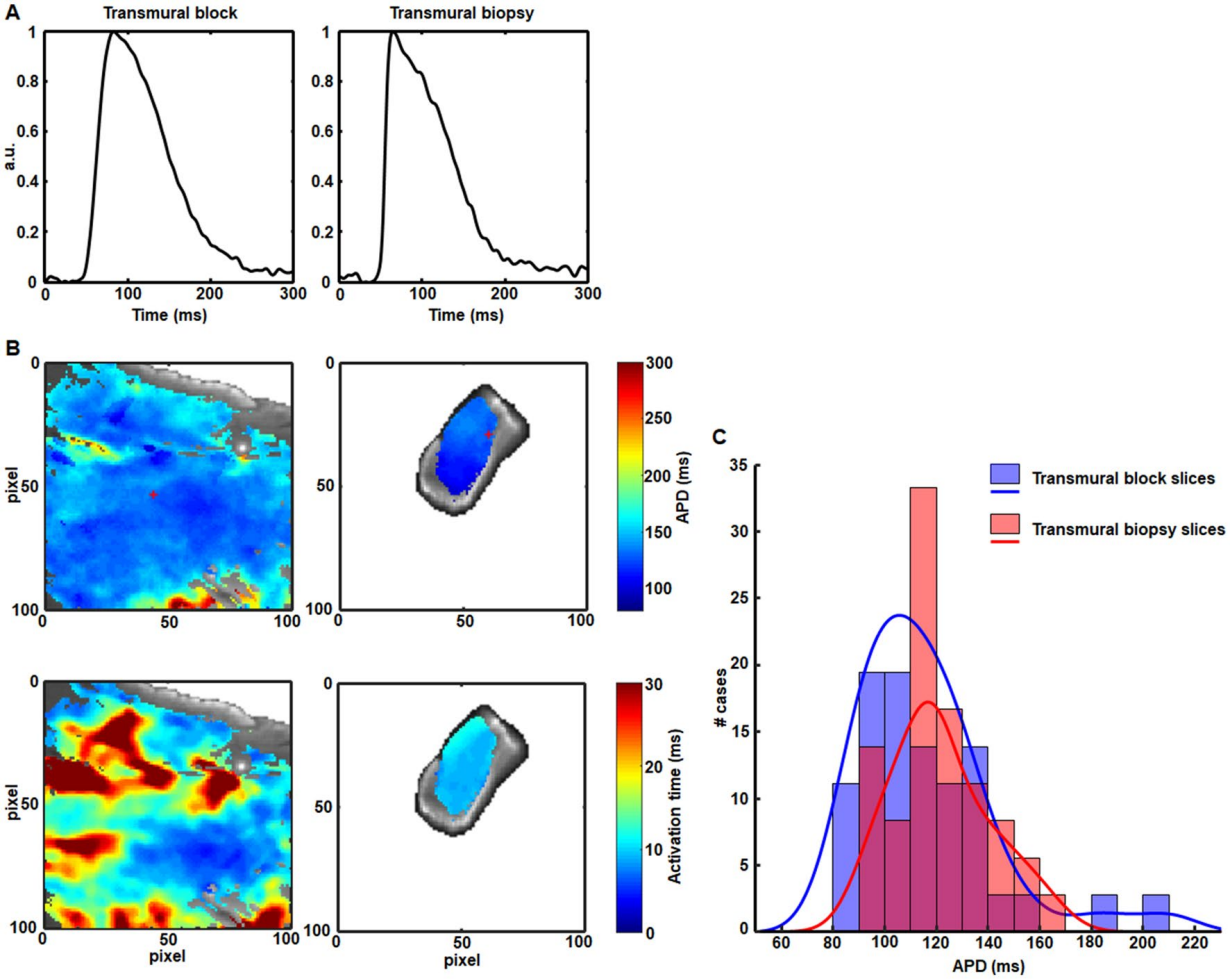
Baseline electrophysiological characterization of transmural tissue block slices and transmural core biopsy slices from pigs. (**A**) Temporally averaged AP for the 20-s transmembrane voltage recording of a point in a tissue block slice (left) and a point in a transmural biopsy slice (right). (**B**) Representative pseudo-color APD and activation time maps at a pacing frequency of 1 Hz for a tissue block slice (left panels) and a transmural biopsy slice (right panels) from the same pig. Crosses indicate the exact pixels for which averaged APs are shown in (**A**). (**C**) Histograms showing the APD distributions (bars) for all tissue block slices (p/n/N = 36/12/4) and all transmural biopsy slices (p/n/N = 36/12/4), along with optimally fitted kernel smoothing functions (lines). Differences between the two APD distributions were not statistically significant according to Mann–Whitney U-test.

**Figure 6:**
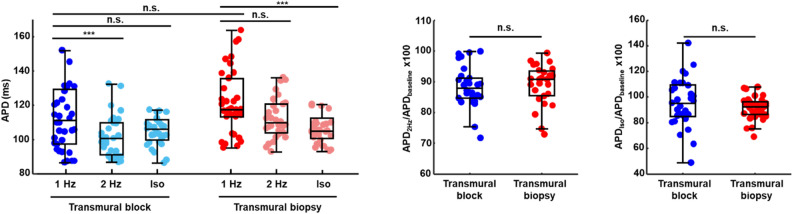
Characterization of the response to an increase in pacing frequency and to β-adrenergic stimulation in pig slices. Left panel: boxplots showing APD in tissue block slices (p/n/N = 36/12/4) and transmural biopsy slices from the same pigs (p/n/N = 36/12/4). Central panel: boxplots showing normalized APD in response to a change in the pacing frequency from 1 to 2 Hz in tissue block slices (p/n/N = 30/10/4) and transmural biopsy slices (p/n/N = 27/9/4) from the same pigs. Right panel: boxplots showing normalized APD in response to 100 nM isoproterenol in tissue block slices (p/n/N = 33/11/4) and transmural biopsy slices (p/n/N = 33/11/4) from the same pigs. ***p < 0.001 and *n.s.* non-statistically significant differences, either in the comparison of APD for 2 Hz versus 1 Hz or isoproterenol versus baseline in the same tissue slices (shown on top of a boxplot) or in the comparison of normalized APD in tissue block slices versus transmural biopsy slices (in between two boxplots) according to Wilcoxon signed rank test.

Optical mapping results in slices from tissue blocks and slices from transmural core biopsies corresponding to the same animal and a nearby location are illustrated in Fig. 5. As can be observed from Fig. 5A, the APs measured in the biopsy preserved the morphology of those measured in the block. This was regularly noted in all tissue specimens from all investigated animals. In addition, the APDs and activation times measured in the biopsies were within the range of APDs and activation times in the blocks, with a more reduced spatial variability in the biopsies due to their smaller volumes, as exemplified in the maps presented in Fig. 5B. The median of APDs in a collection of pixels of all analyzed tissue blocks was 111 ms at 1 Hz pacing frequency. In the biopsy slices the median of APDs was 117 ms (Fig. 6, left panel). Differences in the APD of both types of slices were not statistically significant. Histograms of APD values in the transmural core biopsies and tissue blocks are presented in Fig. 5C. High overlap between both histograms can be observed, without differences in the APD distributions.

Next, the response to an increase in the pacing frequency was evaluated in the porcine transmural core biopsy slices and compared to that in the porcine tissue block slices (Fig. 6, left panel) by the representation of normalized APDs (Fig. 6, central panel). Although the change in APD was only significant for the tissue blocks, similar relative responses were observed in the two cases: median of normalized APDs was 88% in the blocks and 91% in the biopsies, with no statistically significant differences.

Porcine transmural core biopsy slices and tissue block slices were additionally compared in terms of their response to β-adrenergic stimulation (Fig. 6, left and right panel). We observed a change in the APD when applying 100 nM isoproterenol, even if only significant in the case of the biopsies. When comparing the relative differences, normalized APDs did not change between the block slices and the biopsy slices: median normalized APD being 95% in the blocks and 92% in the biopsies.

### Optical mapping of human tissue slices

Viable human slices for electrophysiological analysis were obtained from all papillary muscles and all transmural core biopsies. For each papilla or biopsy, we generally obtained more than 8 slices. 77% of the vibratome slices in the case of the papillary muscles and 58% in the case of the transmural core biopsies rendered electrophysiological signals.

**Figure 7:**
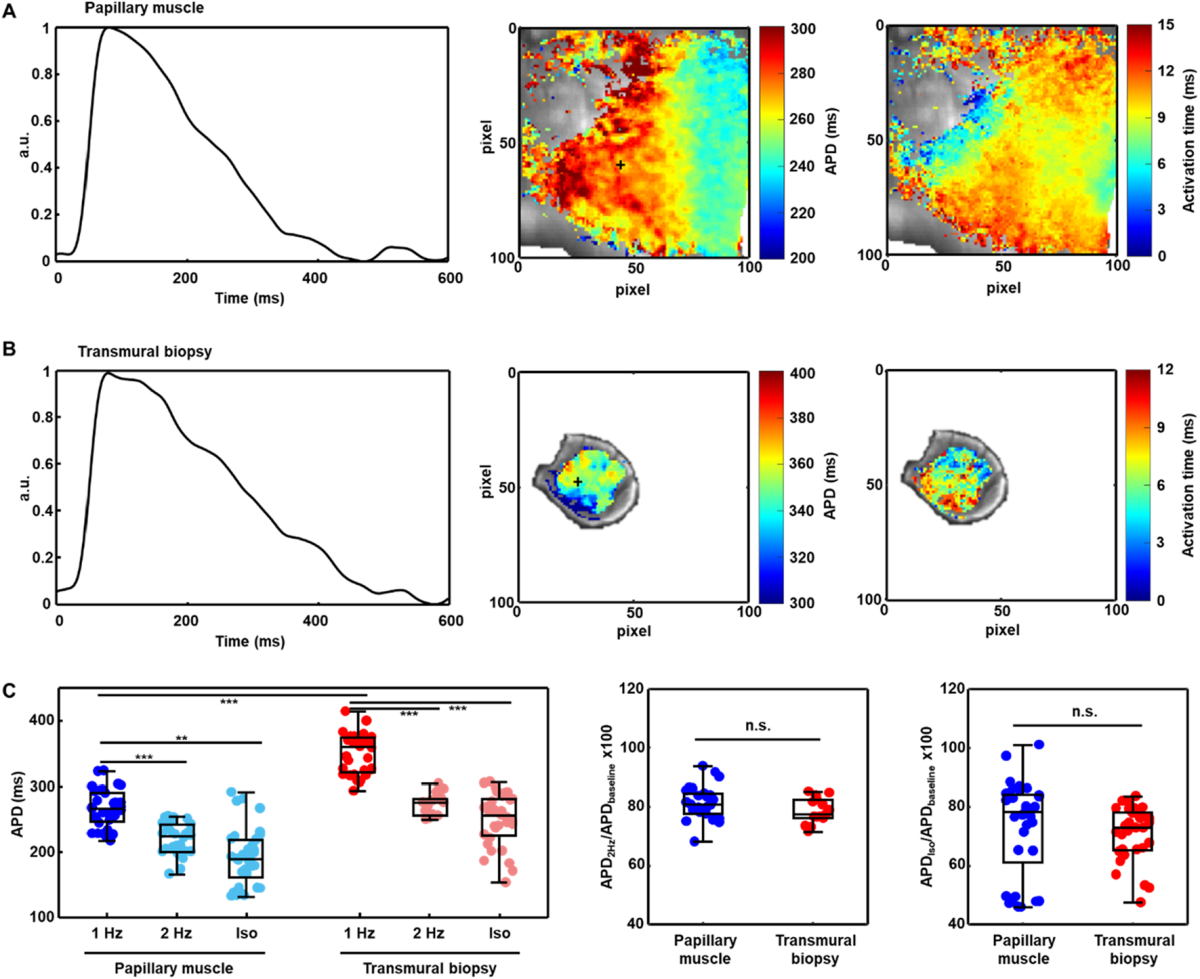
Electrophysiological characterization of human biopsies. (**A**) Temporally averaged AP for the 20-s transmembrane voltage recording of a point in a papillary muscle slice (left) and representative pseudo-color APD and activation time maps at a pacing frequency of 1 Hz for the same papillary muscle slice. The cross indicates the exact pixel for which the averaged AP is shown. (**B**) Same representations as in (**A**) but for a transmural biopsy slice. (**C**) Boxplots showing (left panel) APD in papillary muscle slices (p/n/N = 33/11/4) and transmural biopsy slices (p/n/N = 33/11/4), (central panel) normalized APD in response to a change in the pacing frequency from 1 to 2 Hz in papillary muscle slices (p/n/N = 27/9/4) and transmural biopsy slices (p/n/N = 15/5/4) and (right panel) normalized APD in response to 100 nM isoproterenol in tissue block slices (p/n/N = 33/11/4) and transmural biopsy slices (p/n/N = 33/11/4). **p < 0.01; ***p < 0.001 and *n.s.* non-statistically significant differences, either in the comparison of APD for 2 Hz versus 1 Hz or isoproterenol versus baseline in the same tissue slices (shown on top of a boxplot) or in the comparison of APD at baseline or normalized APD in tissue block slices versus transmural biopsy slices (in between two boxplots), according to Wilcoxon signed rank test (for paired samples) or Mann–Whitney U-test (for unpaired samples).

The results obtained by optically mapping human papillary muscle slices and transmural core biopsy slices are illustrated in Fig. 7. Examples of APs, as well as APD and activation time maps are presented in Fig. 7A for a papillary muscle slice and in Fig. 7B for a transmural biopsy slice. It should be noted that there is no correspondence between the papillary muscle and the biopsy, as these were collected from different patients and locations. Less spatial variability was observed in the biopsy slices as compared to the papillary muscle slices due to their smaller volume. The median APD values in the biopsies from the left ventricular myocardium were larger than in the papillary muscles: median of APD being 266 ms in the papillary muscles and 360 ms in the biopsies (Fig. 7C, left panel).

An increase in the pacing frequency reduced APD in both papillary muscle slices and biopsy slices, as shown in Fig. 7C, left and central panel. The median shortening of APD was 19% in the papillary muscles and 22% in the biopsies.

Similarly, the β-adrenergic agonist isoproterenol reduced APD in papillary muscle slices and biopsy slices (Fig. 7C, left and right panel). The median shortening of APD was 22% in the papillary muscles and 27% in the biopsies.

## Discussion

In this study, we present transmural core biopsies as a safe and widely available method to obtain ventricular tissues during routine cardiac surgery for structural and functional characterization. Although cardiac tissue slices have already been shown to represent a suitable model for physiological and pharmacological studies^6–8,16^, we hereby provide the novelty of obtaining such human tissue slices from small myocardial biopsies. Indeed, biopsies respond similarly to larger pieces of ventricular tissue to increments in the pacing frequency or to β-adrenergic stimulation. This safe and simple method thus represents a breakthrough to increase our understanding of human heart functioning.

Transmural core biopsy slices preserve structural integrity, with predominantly longitudinal alignment of myocardial fibers, regular cross striations and intact tissue structure. In general, we do not observe signs of edema, hypercontracture or myofibril disorganization. Viability of the entire biopsy specimens and vibratome slices, evaluated both by an enzymatic assay and by vital staining imaged with confocal microscopy, is substantially maintained when compared with larger ventricular portions like porcine transmural tissue blocks and human papillary muscles. Only minor injuries are noted on the surface of the biopsy, likely caused by the biopsy needle during the extraction. Upon vibratome cutting, the presence of damaged cells in the tissue slices is mainly restricted to the first outer layers of cells transected during the slicing, which nevertheless presents some viability. Cardiomyocytes' viability increases deep into the tissue slice, in agreement with previous studies on larger ventricular myocardial slices^7,11,12,18,20,33^.

The alignment of the myocardial fibers with the vibratome slicing plane improves viability and restricts tissue damage to the very first cellular layers, as previously reported^6,13,15,16,18,33^. The fiber alignment step is relevant not only to transmural core biopsies, but generally to any other tissue to be sliced. This can explain why the viability of transmural biopsy slices is higher than that of larger tissue slices like those obtained from papillary muscles, which present pronounced tissue heterogeneity and gross areas of chordae tendinae that hinder fiber alignment with the cutting plane. Other indications to increase biopsy slice viability include preservation of the biopsies in very cold solution during transport and slicing^6,7,13,15,16,33^ and fast positioning of the biopsy into agarose to minimize exposure to room temperature. Importantly, a slice thickness of 350 µm is selected to maximize viability while attaining optimal electrophysiological signals. This value is in line with other studies that have reported thicknesses ranging from 150 to 500 µm^11,12,15,37^, with most studies in humans setting it at 300–400 µm^6,7,11,16,33^. Also, a post-cutting recovery time of 30 min is applied, consistent with previously reported times for myocardial slices to allow them to attain steady-state properties^6,7,13,15,16,33,37^.

We note that some transmural core biopsies render slices with areas of not totally aligned fibers, accumulation of fibrosis or presence of blood vessels, which may represent a higher percentage of its total volume as compared to other more commonly used myocardial slices. However, since a set of tissue slices is obtained from each transmural core biopsy, these limitations can be at least partially overcome by the evaluation of other slices from the same biopsy. Indeed, the percentages of pig and human biopsies where we can measure electrophysiological signals, despite being lower than the percentages corresponding to pig transmural tissue blocks and human papillary muscles, are still substantial.

Optical mapping allows the characterization of action potentials from transmural core biopsy slices with high spatial resolution. The electrophysiological evaluation of myocardial tissue slices is evolving from multi-electrode arrays^6,12,15,20,24,33^ to optical mapping for the analysis of action potentials and/or intracellular calcium transients^13,16,19,24,33,37–39^. Here we show that AP duration and morphology are comparable in slices from transmural core biopsies and tissue blocks from the same pig ventricular regions. Median APD values (117 ms and 111 ms in the biopsy and block slices, respectively) are slightly lower than previously published values in experimental works on multicellular preparations^40–44^, which could be explained by the fact that our pig samples come from piglets and young pigs. Indeed, in our APD measures we could observe a trend to shorter APD in those from piglets as compared to those from adult pigs. In any case, the results presented in this study confirm that transmural core biopsy slices mimic the electrical behavior of larger pieces of myocardial tissue and thus constitute an alternative model for cardiac electrophysiological research.

In humans, results from biopsy slices and papillary muscle slices are not comparable, as they are obtained from different patients and, importantly, different ventricular locations. Some but not all the patients were also on antiarrhythmic therapy. Here, median APD in papillary muscle slices paced at 1 Hz frequency is 266 ms. A previous study has reported average APD_90_ values of 320 ms and 443 ms in papillary muscles of healthy and failing hearts respectively, paced at a lower frequency of 0.5 Hz^21^. Both factors, i.e. slower pacing and heart failure-associated remodeling, contribute to APD prolongation. In another study, APD_90_ values of around 290 ms have been reported for ventricular trabeculae and papillary muscles of undiseased organ donors paced at 1 Hz^22^ and APD_90_ values of around 350 ms for papillary slices from failing hearts^15^. Our results on papillary muscle slices obtained from patients with mitral valve disorders are in line with published experimental ranges.

On the other hand, median APD in the human transmural core biopsy slices of this study paced at 1 Hz is 360 ms. This value is in accordance with those reported by other groups for failing and non-failing ventricular tissue preparations. Reported APD values for left ventricular multicellular preparations in the literature range from 275 to 439 ms for non-failing ventricles^16,19,23,38,45–47^ and from 380 to 457 ms for failing ventricles^6,7,16,23,24,38,45,47^. The transmural core biopsies of this study are obtained from patients with coronary artery disease, aortic aneurysm and aortic valve disorders, which can explain the large range of APD values covering from 275 to 475 ms. Morphology of action potentials recorded on transmural biopsies as well as local activation times are in line with those previously reported by other groups^48^.

To the best of our knowledge, this is the first study showing electrophysiological measurements in myocardial slices of the left ventricular free wall from living donors. Since transmural core biopsies can be routinely collected and several slices can be optically mapped, this study sets the basis for future investigations aimed at characterizing inter- and intra-individual variability in the human left ventricle in a large number of individuals. The electrical properties assessed from biopsy slices are those of native myocardium and, thus, they represent a more advanced model than isolated cardiomyocytes. Transmural core biopsies avoid the loss of extracellular matrix and inter-cellular connections as well as the alterations in ion channels associated with cell isolation procedures and provide affordability, ease of use and reproducibility. The use of transmural biopsies can also be useful for cardiac tissue characterization in animal studies without the need to sacrifice the animals and allowing longitudinal research. As several myocardial slices can be obtained from each core biopsy, and more than one biopsy can be obtained from the same animal, multiple conditions can be tested in tissues from the same heart and different time points can be evaluated. This allows us to control for inter-individual bias and to reduce the number of animals required for a given experiment, in line with the 3Rs ethical principles in animal testing. There is already a study in the literature where serial left ventricular needle biopsies have been obtained from dogs undergoing chronic experiments by a trans-thoracic approach that avoids thoracotomy^49^. In that study, the authors show that biopsy sampling does not influence any hemodynamic, mechanical or electrocardiographic parameters while allowing molecular assessment of left ventricular tissue.

Assessment of the response of biopsy tissue slices to an increase in the pacing frequency provides further confirmation of the fact that transmural core biopsies maintain the electrophysiological properties of native myocardium. In pigs, biopsy slices respond to a change in pacing frequency from 1 to 2 Hz with an APD decrease of the same magnitude (median difference below 3%) than the slices obtained from larger tissue blocks of the same ventricular region. In humans, biopsy slices respond to the change in pacing frequency with a more remarkable decrease in APD than that observed in pigs, but in any case of the same magnitude than slices from human papillary muscles (median difference below 4%). The observed APD reduction following an increment in pacing frequency is in agreement with previously reported outcomes for other pig and human multicellular preparations^6,16,23,46,47^.

The physiological responsiveness of transmural core biopsy slices is additionally substantiated by assessing the response to the administration of the β-adrenergic agonist isoproterenol. Porcine slices from transmural core biopsies and tissue blocks respond equally to β-adrenergic stimulation, with no statistically significant differences in the APD decrease (less than 3% median difference). Similarly happens with human slices from transmural core biopsies and papillary muscles, which respond more notably than pig tissues, but similarly one to another (less than 6% median difference, no statistically significant differences). The β-adrenergic stimulation-induced APD reduction measured in the pig and human tissue preparations of this study are in line with other studies of the literature^7,16,20,38,50,51^. All presented results support the suitability of transmural biopsy slices for studies of healthy and diseased human ventricular tissue.

Some limitations and future extensions of this work are as follows. Due to the small size of human ventricular transmural core biopsies, imposed by safety considerations, the percentage of damaged slices from each biopsy specimen is larger than for other multicellular preparations. Nevertheless, since a large number of slices can be obtained from each biopsy and more than one biopsy can be obtained from each donor, it is still possible to perform different types of analysis in a relatively high proportion of donors.

We have characterized APD and activation times in mid-myocardial slices from transmural core biopsy slices from pig and humans and we have shown that, despite the reduced size of the biopsies, they still allow evaluation of spatial AP heterogeneities. Future studies could additionally investigate other electrophysiological properties from transmural core biopsies, such as conduction velocity, refractory period or restitution curves, both at baseline and in response to pharmacological treatments. Also, other studies could characterize differences in the electrophysiology of different regions (epicardium, mid-myocardium, endocardium) within the myocardial wall of each transmural core biopsy, which would help to investigate the effects on transmural heterogeneity under diseased states, including inherited channelopathies. Moreover, transmural core biopsy slices could be used to understand depolarization abnormalities, like those observed in Brugada syndrome, while accounting for myocardial tissue heterogeneity.

In conclusion, we present and validate transmural core biopsies as a novel, safe, and handy procedure to obtain human left ventricular tissue from living donors. Slices from transmural core biopsies preserve the structural and functional properties of larger pieces of myocardial tissue and respond similarly to changes in pacing frequency and β-adrenergic stimulation. This study opens the door to future investigations aimed at characterizing cardiac behavior in healthy and diseased hearts from a large number of individuals.

## Supplementary information

Supplementary Information.
