# Supplementary material for: Minimally invasive system to reliably characterize ventricular electrophysiology from living donors
Source: Sci Rep. 2020 Nov 17;10:19941. doi: 10.1038/s41598-020-77076-0 (PMC7673124; doi:10.1038/s41598-020-77076-0)
Supplement: Supplementary file 1 — Supplementary Information. [file 41598_2020_77076_MOESM1_ESM.docx]

**Minimally invasive system to reliably characterize ventricular electrophysiology from living donors**

Aida Oliván-Viguera, PhD (1), María Pérez-Zabalza, PhD (1), Laura García-Mendívil MSc (1), Konstantinos A. Mountris PhD (1), Sofía Orós-Rodrigo MSc (1), Estel Ramos-Marqués PhD (1), José María Vallejo-Gil MD (2), Pedro Carlos Fresneda-Roldán MD (2), Javier Fañanás-Mastral MD (2), Manuel Vázquez-Sancho MD (2), Marta Matamala-Adell MD, PhD (2), Fernando Sorribas-Berjón MD (2), Javier André Bellido-Morales MD (2), Francisco Javier Mancebón-Sierra MD (2), Alexánder Sebastián Vaca-Núñez MD (2), Carlos Ballester-Cuenca MD (2), Miguel Ángel Marigil MD (3), Cristina Pastor MD (4), Laura Ordovás PhD (1,5), Ralf Köhler PhD (4,5), Emiliano Diez MD, PhD (6), Esther Pueyo PhD (1,7)

(1) Biomedical Signal Interpretation and Computational Simulation (BSICoS), Aragón. Institute of Engineering Research (I3A), University of Zaragoza & Instituto de Investigación Sanitaria (IIS) Aragón, Zaragoza, Spain
(2) Department of Cardiovascular Surgery, University Hospital Miguel Servet, Zaragoza, Spain

(3) San Jorge Hospital, Department of Pathology, Huesca, Spain
(4) Aragón Institute of Health Sciences (IACS), Zaragoza, Spain
(5) Aragón Agency for Research and Development (ARAID), Zaragoza, Spain
(6) Institute of Experimental Medicine and Biology of Cuyo (IMBECU), CONICET, Mendoza, Argentina
(7) Biomedical Research Networking Center in Bioengineering, Biomaterials and Nanomedicine (CIBER-BBN), Zaragoza, Spain

**SUPPLEMENTARY MATERIAL**

**Supplementary Figures**


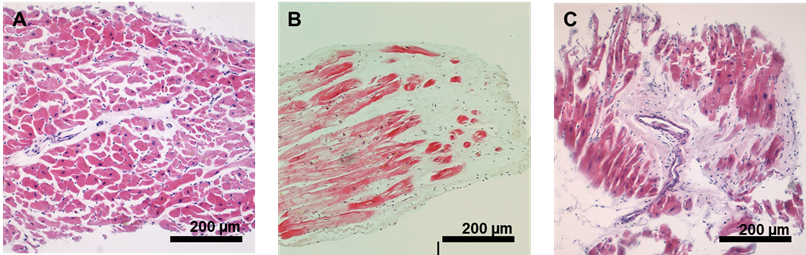


**Supplementary Figure 1**: **Histological evaluation of tissue slices with transverse fibers or major areas devoid of cardiomyocytes**. Representative photomicrographs stained with hematoxylin/eosin and imaged at 10x magnification. A) A transmural biopsy slice with a large area of transverse fibers as a result of incorrect alignment of the biopsy during vibratome slicing. B) A human papillary muscle slice with a high degree of intercalated connective tissue of the chordae tendinae. C) A transmural core biopsy slice with a large area occupied by a coronary artery and its surrounding connective tissue.

**Supplementary Tables**

**Supplementary Table 1: Clinical characteristics of patients whose tissues were employed for optical mapping characterization.**

| Sex | Age | Disease | Antiarrhythmic therapy |
| --- | --- | --- | --- |
| Transmural core biopsy | | | |
| m | 63 | Three-vessel coronary artery disease | Bisoprolol |
| m | 73 | Valvular cardiomyopathy | No |
| m | 72 | Aortic valve insufficiency | Bisoprolol |
| f | 74 | Aneurysm of the aorta, aortic valve insufficiency | No |
| Papillary muscle | | | |
| f | 60 | Mitral valve insufficiency | Bisoprolol |
| m | 68 | Mitral valve insufficiency | No |
| f | 77 | Mitral and tricuspid valvulopathy | Bisoprolol and Digitalis |
| m | 69 | Mitral valve stenosis | Bisoprolol |
| m (male), f (female) | | |  |

**Expanded Methods**

1. *Myocardial tissue collection*

Porcine left ventricular transmural biopsy specimens were obtained from 5 white pigs (*Sus scrofa domesticus*) of 4-7 weeks of age (n=3) or 18-22 weeks of age (n=2) at the Experimental Surgery Service of the Aragon Health Sciences Institute, after the animals were necessarily sacrificed at the end of other research studies. Specimens were collected following euthanasia by intravenous administration of potassium chloride solution (1 mEq / Kg) performed under deep anesthesia with propofol (intravenous administration, up to 6 mg/Kg) and inhaled sevoflurane (1.9%). A disposable 14 G tru-cut biopsy needle (Bard Mission 1410MS, Bard) with semi-automatic firing and a fixed penetration depth of 10 mm was used to extract biopsies of approximately 1.2 mm diameter and up to 10 mm long (Figure 1). Additionally, transmural tissue blocks (surface area ≈ 5 x 7 mm) were cut with a single edge razor blade from a neighboring zone (Figure 1). All animal experiments complied with the regulations of the local animal welfare committee for the care and use of experimental animals and were approved by local authorities (Ethics Committee on Animal Experimentation, CEAEA, of the University of Zaragoza). All animal procedures conformed to the guidelines from Directive 2010/63/EU of the European Parliament on the protection of animals used for scientific purposes.

Human left ventricular transmural core biopsy specimens were collected by experienced cardiothoracic surgeons of the Cardiovascular Surgery Department at Miguel Servet University Hospital. Specimens were obtained from 21 patients of both sexes, with ages ranging from 46 to 78 y.o., undergoing valve replacement surgery or coronary artery bypass grafting. A disposable 14 G tru-cut biopsy needle (Bard Mission 1410MS, Bard) was used to extract the biopsy during cardiac arrest soon after the patient was placed on cardiopulmonary bypass. One transmural core biopsy was obtained from every patient from the lateral wall of the left ventricle, near the base of the heart, on a site with no evidence of ischemia or any other macroscopic pathology ^1^. As in previous studies, the biopsy site was oversewn and no complications have been noted to date ^2–4^. Additionally, papillary muscles resected during valve replacement from 8 different patients of both sexes, with ages ranging from 59 to 78 y.o., were included for comparison. All patients gave written informed consent before surgery and prior to their inclusion in the study. The study conforms to the principles outlined in the Declaration of Helsinki and was approved by the local Ethics Committee (CEICA, reference number PI17/0023).

1. *Tissue slice preparation*

Upon collection, porcine and human tissues were immediately submerged in ice cold pre-oxygenated (gassed with pure oxygen), freshly prepared, cold Tyrode’s solution (composition in mM: NaCl 140, KCl 6, CaCl_2_ 1.8, MgCl_2_ (·6H_2_O) 1, glucose 10, HEPES 10) supplemented with 30 mM 2, 3-butanedione monoxime (BDM) to inhibit contractile activity, pH adjusted to 7.4, all chemicals from Sigma Aldrich. Transport time to the laboratory was less than 10 minutes for human tissues and less than 1 hour for porcine tissues.

Tissue blocks were directly glued to the vibratome cutting stage, using cyanoacrylate instant glue (Loctite, Henkel, Spain), mounting them epicardium-side down to ensure maximum longitudinal alignment of muscle fibers with the slicing plane (Figure 1). This was based on the fact that myocardial fascicles mainly wrap around the ventricular lumen so that the dominant fiber orientation runs parallel to the epicardium ^5–7^ . This alignment step is fundamental to reduce the number of fibers transected during sectioning and, thus, to minimize tissue damage.

In the case of the transmural core biopsies, these were kept upright to be sliced parallel to the epicardial plane by embedding them in low-melting agarose (Roth, Karlsruhe, Germany), dissolved at 4% in Tyrode’s solution, at a temperature below 30°C (near to the gelling temperature of 28 °C) and quickly chilled on an ice bath to allow agarose to solidify. Agarose blocks containing the biopsy where then rapidly glued onto the vibratome cutting stage, with the biopsy in upright position (Figure 1) and immediately transferred to the cutting bath.

For papillary muscles, due to its heterogeneous morphology, correct orientation of the fibers was challenging and wrong positioning was found to be a cause of poor slice viability. Success was maximized when papillary muscles were sliced with the chordae tendinae aligned with the slicing plane. When necessary, papillary muscles were embedded in low-melting agarose to keep them in the correct position for slicing.

350-μm-thick slices from tissue blocks, transmural core biopsies and papillary muscles were cut in ice-cold (4ºC) pre-oxygenated 30 mM BDM-Tyrode’s solution. The 350-μm thickness was chosen to facilitate oxygen diffusion to all cells in the slice while allowing measurement of fluorescent signals ^8–10^. A high precision vibratome (Leica VT1200S, Leica Microsystems, Germany) with stainless steel blades (Astra Superior Platinum double edge razor blades), advancing at a low speed of 0.03 mm/s and oscillating at an amplitude of 2 mm and a vibration frequency of 80 Hz, was used. Calibration was performed before slicing each biopsy to have an out-of-plane blade deviation lower than 0.5 µm. Endocardial trabecular layers from porcine transmural blocks, gross chordae tendinae areas from human papillary muscles as well as the region located approximately 1.5 mm from the epicardial side of transmural biopsies were all trimmed to obtain myocardial slices for further analysis.

After slicing, sections were paraformaldehyde fixed for histological evaluation, stained for viability assessment or kept at room temperature in pre-oxygenated Tyrode’s solution for electrophysiological analysis by optical mapping within the following eight hours.

1. *Histological staining*

To assess preservation of the morphology and structure of cardiac tissue after vibratome sectioning, pig and human biopsy slices were fixed in 4% paraformaldehyde for 30 minutes at 4°C and subsequently stored in PBS with 0.01% Na-azide at 4°C until paraffin embedding. Thereafter, paraffin-embedded tissue was cut in 3-5 µm-thick sections using a microtome (Reichert-Jung Mod 1130, Biocut) and stained with hematoxylin/eosin or Masson's trichrome. During sectioning, slices were kept in upright position, allowing the generation of cross-sections in the same plane as the vibratome slice. Stained sections were imaged with a Leica DM5000B microscope (Leica Microsystems, Germany) at 20x and 63x magnification.

1. *Viability assessment by TTC assay*

To quantify the vitality of the cardiac tissues from the moment of collection until its sectioning in the vibratome, triphenyltetrazolium chloride (TTC) staining, a well-established, fast and macroscopic viability test commonly employed for infarct size delineation in experimental cardiology, was performed ^11^. TTC assay is based on the enzymatic activity of mitochondrial dehydrogenases, which in the presence of NADH reduce a pale yellow dye to a brick-red precipitate, thus staining viable myocardium in red. Porcine tissue blocks and transmural core biopsies as well as human papillary muscles and transmural core biopsies were incubated in 2% TTC solution in Tyrode’s buffer for 30 minutes at 37°C. Dead tissue specimens obtained by exposing transmural biopsies to DMSO for 30 minutes before TTC staining were used as negative controls.

TTC assay was additionally applied to slices from tissue blocks, papillary muscles and transmural core biopsies to assess whole slice vitality following vibratome sectioning and electrophysiological assessment. DMSO-treated slices were considered as negative controls. The dye was subsequently extracted with 95% ethanol during 30 minutes. The absorbance of the supernatant was measured at 485 nm in a spectrophotometer (Synergy HT, Biotek) and values were corrected for total tissue weight ^12,13^. Absorbance values were expressed as relative to the values measured for negative controls (i.e. after subtraction of the value for the corresponding negative control tissue). Normalized absorbance values were calculated as percentages of the maximum absorbance value corresponding to each type of sample (i.e. after dividing by the value of the corresponding positive control).

1. *Viability assessment by viable/damaged assay and confocal microscopy*

Viability of the surface of vibratome slices was additionally assessed using viable/damaged staining and confocal microscopy. Identification of viable/damaged cells was performed by vital staining with Syto9 (stains green the nuclei of all cells) and Dapi (stains blue the nuclei of damaged cells). Immediately upon being cut, slices were loaded with 10 µM Syto9 (Invitrogen) and 10 µg/ml Dapi (Invitrogen), in Tyrode’s solution at room temperature for 30 minutes, and observed under a confocal laser scanning microscope LSM 880 (Zeiss) equipped with dry (10x) objective lens. Dapi was excited with a 405 nm laser and emitted fluorescence was filtered at a wavelength of 410-495 nm, whereas Syto9 was excited with a 488 nm argon laser and its emission was filtered at 495-634 nm. Z-series of images were collected at 50-150 longitudinal focal planes, with the optimal confocal plane thickness for the objective employed. Due to the high cellular density of the cardiac tissue, laser penetration is limited to 30-40 µm and therefore the evaluation of the viability with this method is approximately restricted to the first two layers of cells in the surface of the preparation. The central core of both sides of the tissue slices were analyzed.

Images were initially processed using Zen2.3-Blue software (Zeiss). Z-stacks from each side of the slice were divided into an external and an internal subset and each of them was compressed into a 2D image to evaluate the percentage of damaged cells in the first half and second half portions of those outer layers of cells. Custom-written software (MATLAB R2017a, The MathWorks Inc., Natick, MA) was developed for image processing. Specifically, the compressed 2D images of the internal and external Z-stacks were analyzed by a five-step procedure. First, gamma correction was applied to the green channel of the compressed RGB images to increase the contrast of the regions with viable cells nuclei. The value for the gamma coefficient was selected in the range 1-1.8. Subsequently, grey-level images for viable and damaged cells nuclei were obtained by extracting the green and blue channel of the compressed image, respectively. The grey-level images were converted to binary masks by using a binarization filter, with a binarization threshold of 0.2-0.5 applied to remove any binarization artefacts from the thresholded images. Finally, the centroid locations of the bright regions were extracted to calculate the number of nuclei in each binary image. The percentage of viable cells nuclei (VNP) was calculated as the ratio of the number of viable cells nuclei centroids over the number of total (viable and damaged cells) nuclei centroids:

$$VNP=\frac{\# viable centroids}{\left( \# viable centroids +\# damaged centroids \right)}x 100\%$$

1. *Optical mapping of transmembrane potential*

Myocardial tissue slices were optically mapped with a high speed and low noise optical mapping system consisting of a MiCAM O5-Ultima CMOS camera (SciMedia, Costa Mesa, CA) with a spatial resolution of 100x100 pixels, with 100x100 µm per pixel.

Transmembrane potential was measured using the voltage-sensitive dye RH237 (Invitrogen, Carlsbad, CA), which was loaded onto the tissue and allowed to incubate for 15 minutes at room temperature and a concentration of 7.5 µM in pre-oxygenated Tyrode’s solution. To suppress motion artefacts, the excitation-contraction uncoupler blebbistatin (10 µM, Tocris Bioscience, St. Louis, MO) was dispensed and incubated for 30 minutes prior the staining process. Stock solutions of RH237 were prepared in DMSO at a concentration of 1.25 mg/ml and stored in the dark at -20°C until use. Blebbistatin was prediluted to 2 mg/ml in DMSO and stored in the dark at 4°C. After staining, tissue slices were washed in pre-oxygenated Tyrode´s solution. Optical measurements of transmembrane potential were conducted in Tyrode’s solution at 35°C, with the solution in the tissue chamber being pre-oxygenated to maintain oxygen content.

Tissue slices were excited using light from a high intensity LED illumination system through an excitation filter of 530 nm (LEX2-LZ4, SciMedia, Costa Mesa, CA, USA). Emitted fluorescence was collected through a THT-macroscope (SciMedia) by a 2x magnification objective lens (Leica, Germany) and detected with a camera equipped with a 715 nm long-pass filter. All optical signals were recorded with a temporal resolution of 1 ms.

Tissue slices were stimulated while placed in a heated chamber (RC-27NE in a PM-6 heated platform, Warner Instruments) equipped with two platinum field-stimulation electrodes. By using a CS-20 stimulator (Cibertec, Spain), 40 V monopolar pulses of 0.5 ms duration were delivered at pacing frequencies of 1 and 2 Hz. 20-second recordings were acquired after a short period of stimulation to allow slices to adjust to the pacing rate.

β-adrenergic stimulation responsiveness was evaluated by application of isoproterenol hydrochloride (100 nM, Sigma Aldrich) slowly washed into the bath solution. Stock solutions of isoproterenol 10 µM were prepared in distilled water and stored in the dark at -20°C for up to one month.

1. *Optical mapping data analysis*

Action potential (AP) signals from 24 porcine slices (12 from transmural cubes and 12 from transmural core biopsies of four pigs) and 22 human slices (11 from papillary muscles of four patients and 11 from transmural core biopsies of four other patients) were evaluated. Human transmural core biopsy slices were from a group of four patients whose age ranged from 63 to 74 y.o., whereas human papillary muscle slices were from a different group of four patients whose age ranged from 60 to 77 y.o.

Optical AP signals were high-pass filtered (0.04 Hz cut-off frequency) to remove baseline drift and subsequently filtered by an adaptive spatio-temporal Gaussian filter ^14^. AP duration (APD) was calculated by measuring the elapsed time between the activation, defined as the time occurrence of the maximum AP upslope, and the time for 80% repolarization. APD and activation time maps of the myocardial slices were generated for the whole set of pixels ^15^. A signal-to-noise ratio (SNR) value was calculated for each of them as the AP amplitude divided by the root mean-square of voltage during the diastolic interval ^16^. A threshold on SNR was set and APD and activation maps were presented only for pixels with SNR above it.

Relative APD values measured after β-adrenergic stimulation or after increasing the stimulation frequency to 2 Hz were calculated as normalized with respect to those measured at baseline while pacing at 1 Hz.

1. *Statistical analysis*

Quantitative data are presented as median [interquartile range (IQR): Q3 – Q1] or as averaged values for percentages of cases. The notation n/N is used to denote n slices from N tissue blocks/papillary muscles or transmural core biopsies. In the analysis of viability evaluation by confocal microscopy imaging, the notation i/n/N denote i images of different areas from n slices from N tissue blocks, papillary muscles or transmural core biopsies. When optical mapping measurements are presented from different pixels across each slice, the notation p/n/N is used to denote p pixels from n slices from N tissue blocks or transmural core biopsies.

The effects of β-adrenergic stimulation and of increased pacing frequency on APD were assessed by using the non-parametric Wilcoxon signed rank test for paired samples, as the data were not normally distributed according to Shapiro-Wilk test. To compare normalized APD values between the groups of measurements from tissue blocks/papillary muscles and the group of measurements from transmural core biopsies, the non-parametric Mann-Whitney U-test for unpaired samples was used.

A p-value <0.05 was considered as statistically significant.

**References**

1. Campbell, D. J. *et al.* Diastolic Dysfunction of Aging Is Independent of Myocardial Structure but Associated with Plasma Advanced Glycation End-Product Levels. *PLoS One* **7**, 1–14 (2012).

2. Singh, A. K., Farrugia, R., Teplitz, C. & Karlson, K. E. Electrolyte versus Blood Cardioplegia: Randomized Clinical and Myocardial Ultrastructural Study. *Ann. Thorac. Surg.* **33**, 218–227 (1982).

3. Ruifrok, W. T. *et al.* Apoptosis during CABG surgery with the use of cardiopulmonary bypass is prominent in ventricular but not in atrial myocardium. *Netherlands Hear. J.* **18**, 236–242 (2010).

4. Vassiliou, V. S. *et al.* Identification of myocardial diffuse fibrosis by 11 heartbeat MOLLI T 1 mapping: averaging to improve precision and correlation with collagen volume fraction. *Magn. Reson. Mater. Physics, Biol. Med.* **31**, 101–113 (2018).

5. Plank, G. *et al.* Generation of histo-anatomically representative models of the individual heart: Tools and application. *Philos. Trans. R. Soc. A Math. Phys. Eng. Sci.* **367**, 2257–2292 (2009).

6. Stephenson, R. S. *et al.* High resolution 3-Dimensional imaging of the human cardiac conduction system from microanatomy to mathematical modeling. *Sci. Rep.* **7**, 7188 (2017).

7. LeGrice, I. J. *et al.* Laminar structure of the heart: ventricular myocyte arrangement and connective tissue architecture in the dog. *Am. J. Physiol.* **269**, H571-82 (1995).

8. Bussek, A. *et al.* Tissue Slices from Adult Mammalian Hearts as a Model for Pharmacological Drug Testing. *Cell. Physiol. Biochem.* **24**, 527–536 (2009).

9. Camelliti, P. *et al.* Adult human heart slices are a multicellular system suitable for electrophysiological and pharmacological studies. *J. Mol. Cell. Cardiol.* **51**, 390–398 (2011).

10. Wang, K. *et al.* Cardiac tissue slices: Preparation, handling, and successful optical mapping. *Am. J. Physiol. - Hear. Circ. Physiol.* **308**, H1112–H1125 (2015).

11. Ito, W. D. *et al.* Infarct size measurement by triphenyltetrazolium chloride staining versus in vivo injection of propidium lodide. *J. Mol. Cell. Cardiol.* **29**, 2169–2175 (1997).

12. Thomas, R. C. *et al.* A Myocardial Slice Culture Model Reveals Alpha-1A-Adrenergic Receptor Signaling in the Human Heart. *JACC: Basic to Translational Science* **1**, 155–167 (2016).

13. Ou, Q. *et al.* Physiological Biomimetic Culture System for Pig and Human Heart Slices. *Circ. Res.* **125**, 628–642 (2019).

14. Pollnow, S. *et al.* An adaptive spatio-temporal Gaussian filter for processing cardiac optical mapping data. *Comput. Biol. Med.* **102**, 267–277 (2018).

15. O’Shea, C. *et al.* ElectroMap: High-throughput open-source software for analysis and mapping of cardiac electrophysiology. *Sci. Rep.* **9**, 1–13 (2019).

16. Mironov, S. F., Vetter, F. J. & Pertsov, A. M. Fluorescence imaging of cardiac propagation: spectral properties and filtering of optical action potentials. *Am. J. Physiol. Circ. Physiol.* **291**, H327–H335 (2006).
